# Case report: Prenatal diagnosis of Ectrodactyly–Ectodermal dysplasia–Cleft syndrome (EEC) in a fetus with cleft lip and polycystic kidney

**DOI:** 10.3389/fgene.2022.1002089

**Published:** 2022-10-31

**Authors:** He Biwei, Su Min, Wang Yanlin, Zhao Xinrong, Gao Li, Hua Renyi, Sun Jinling, Wang Shan, Wu Yi, Cheng Weiwei

**Affiliations:** ^1^ Prenatal Diagnostic Center, International Peace Maternity and Child Health Hospital, School of Medicine, Shanghai JiaoTong University, Shanghai, China; ^2^ Shanghai Key Laboratory of Embryo Original Disease, Shanghai, China; ^3^ Department of Reproductive Genetics, International Peace Maternity and Child Health Hospital, School of Medicine, Shanghai Jiao Tong University, Shanghai, China

**Keywords:** EEC, p63, prenatal phenotypic spectrum, genitourinary anomalies, whole-exome sequencing

## Abstract

Ectrodactyly–ectodermal dysplasia–cleft (EEC) syndrome is an autosomal dominant disorder characterized by ectrodactyly, ectodermal dysplasia, and orofacial clefting. Reduced penetrance is manifested in these core features and additional under-recognized features, especially in prenatal cases. Here, we present a fetus with EEC syndrome at 22 weeks gestation, in which the cleft lip and palate and the right polycystic kidney are shown by prenatal ultrasound. A *de novo* missense mutation of R304W in the *TP63* gene is confirmed by whole-exome sequencing associated with EEC syndrome. We further investigate the reported *TP63*-related prenatal cases and provide a more complete picture of the prenatal phenotypic spectrum about EEC. It illustrates the potential severity of genitourinary anomalies in *TP63*-related disorders and highlights the need to counsel for the possibility of EEC syndrome, given the occurrence of genitourinary anomalies with orofacial cleft or limb deformities.

## Introduction

Ectrodactyly–ectodermal dysplasia–cleft (EEC, OMIM 604292) syndrome is a rare, autosomal dominant syndrome characterized by 1) ectrodactyly or lobster-claw deformity, 2) ectodermal dysplasia (anomalies in hair, teeth, nail, skin, sweat gland, lacrimal duct, breast, and nipple development), and 3) cleft lip and/or cleft palate. Additional clinical features have also been described: genitourinary and external ear malformations, hearing loss, chronic respiratory infections, ventricular cardiomyopathy, and developmental delay (Yin et al., 2010; Goldsmith et al., 2014; Zheng et al., 2019). There is a wide range of variability in clinical manifestations with occasional non-penetrance.

The tumor protein p63 gene (*TP63*) mutations account for most and possibly all cases of classical EEC syndrome, which is located on chromosome 3q27 and encodes a transcription factor homologous to the tumor suppressors p53 and p73 (Ianakiev et al., 2000; Barrow et al., 2002; Bertola et al., 2004; Mikkola, 2007; Yin et al., 2010). Genomic organization of TP63 is complexed with at least six different isoforms (Yin et al., 2010). TP63 mutations result in amino acid substitutions in the DNA-binding domain common to all known p63 isoforms (Brunner et al., 2002). The five arginine codons Arg204, Arg227, Arg279, Arg280, and Arg304 are the most frequently mutated residues, which commonly affect CpG sites, accounting for about 80% of EEC syndrome cases (Barrow et al., 2002; Yin et al., 2010).

Up to now, over 200 cases of EEC syndrome have been reported in the literature, whereas almost all of them were postnatal cases. Prenatal data concerning the EEC syndrome are still very limited. The prenatal detection of EEC syndrome depends on which features can be detectable by ultrasound. There are few literature reports available on antenatal ultrasound findings in patients with EEC; furthermore, only a few studies have focused on prenatal findings in patients with genitourinary anomalies. Consequently, genitourinary anomalies remain a common but under-recognized feature of EEC syndrome (Hyder et al., 2017).

Recently, whole-exome sequencing (WES) has provided the opportunity for molecular genetic screening of rare human diseases. Here, we report a fetus with cleft lip and palate and right polycystic kidney and identify a pathogenic variation of the *TP63* gene by WES associated with EEC syndrome. We also summarize the prenatal ultrasound phenotypes caused by TP63 mutations. Prenatal characteristics of the TP63 mutations are determined to extend the prenatal phenotypes of EEC and improve the accuracy of prenatal diagnosis.

## Materials and methods

### Patient samples

A woman was referred to our prenatal diagnosis center due to suspicious fetal structural anomalies. Ultrasound showed an enlarged bladder during the first trimester screening scan for chromosomal anomalies. Chorionic sampling could not be performed due to the posterior placenta. The woman accepted amniocentesis and chromosome microarray analysis (CMA) as the first-line genetic testing at 17 weeks. After 3 weeks, the CMA and karyotyping results were both normal. However, the routine fetal anatomy scan showed cleft lip and right kidney dysplasia. Parents decided to terminate the pregnancy by the intra-amniotic injection of rivanol solution after signing the informed consent, and they accepted trio whole-exome sequencing (trio-WES) to identify the underlying etiology. Autopsy was recommended to obtain more detailed phenotypic information. The study was approved by the Medical Ethics Committee of the International Peace Maternity and Child Health Hospital (GKLW 2019-24).

### Chromosomal microarray analysis

CMA was performed using the Affymetrix CytoScan 750K Array (Affymetrix, Inc., Santa Clara, CA, United States), and CNVs were determined by Affymetrix Chromosome Analysis Suite software 3.2 (Affymetrix, Inc., Santa Clara, CA, United States). The pathogenicity of CNVs was evaluated under the technical standards of the American College of Medical Genetics and Genomics and the Clinical Genome Resource in 2019.

### Whole-exome sequencing

Peripheral blood of the trio was collected; then, the DNA was extracted and purified. The targeted TP63 exons and flanking regions were captured by the customized clinical exome chip (BGI V4). The library was built under the manufacturer’s recommendations and sequenced on BGI MiSeq 2000. Over 150 M of raw sequences were generated, resulting in an average depth of 150X in the target region and 95% regions with coverage >30X. Low-quality and adapter reads were removed using Trimmomatic (Trimmomatic.0.39) (Bolger et al., 2014). The remained high-quality reads were aligned to the human genome GRCh37/hg19 with the Burrows–Wheeler Aligner (Li and Durbin, 2010). Genome Analysis Toolkit 4 (Van der Auwera et al., 2013) was used to identify short variants. The duplicates were marked by the MarkDuplicates function, base quality scores were recalibrated by the BQSR function, and finally, the variants were called by the HaplotypeCaller algorithm. Candidate events were displayed using the Integrative Genomics Viewer (IGV); the position and quality of variants, as well as strand orientation, were checked carefully.

### Sanger sequencing

PCR primers were designed to amplify exon 8 of TP63. The mutation of the proband was sequenced from a 640-bp DNA fragment amplified using the primer pair 5′-CTG​GTA​GTA​CGT​TGG​CGA​TG-3′ and 5′-ATA​AGG​AGG​TGG​AAG​GAT​GG-3’. Sanger sequencing was performed using the ABI 3730xl DNA automated sequencer (Applied Biosystems, Foster City, CA, United States).

### Case presentation

**FIGURE 1:**
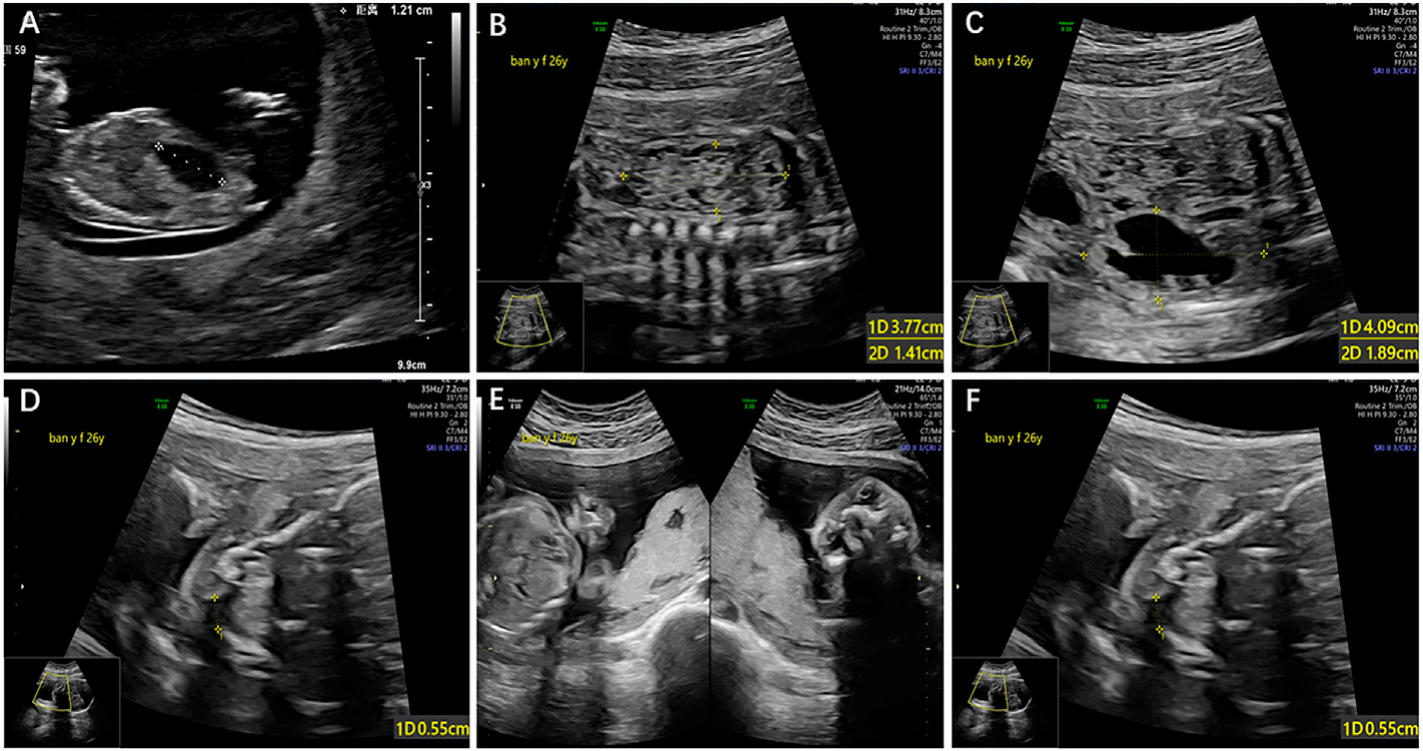
**(A)**: Enlarged bladder with the diameter of 12 mm, **(B)** right multicystic kidney, **(C)** left hydronephrosis with the width of 4 cm, **(D)** cleft palate, **(E)** and **(F)**cleft lip.

A 35-year-old G2P0 woman at the gestational age of 11 + 6 was referred to the prenatal diagnosis center of the International Peace Maternity and Child Health Hospital, due to an enlarged fetal bladder. The ultrasound showed a normal nuchal translucency (NT = 1.3 mm) and an enlarged bladder with the diameter of 12 mm (Figure 1A). It was her secondary pregnancy after one ectopic in 2019. The couple was not consanguineous, and the family history was unremarkable for any other known congenital anomalies or otherwise. After 1 week, a repeated ultrasound showed a slightly reduced bladder with a diameter of 10 mm. Considering the advanced maternal age and contingency of ultrasound findings, chorionic villus sampling (CVS) was then suggested. However, CVS could not be performed due to the posterior placenta. Non-invasive prenatal testing (NIPT) was conducted at 15 weeks of gestation, and the NIPT result was negative. At 17 weeks, the woman accepted amniocentesis and chromosome microarray analysis (CMA) as the first-line genetic testing. After 3 weeks, the CMA and karyotyping results were both normal. At 22 weeks, the routine fetal anomaly scan showed multiple structural abnormalities including the right multicystic kidney, left hydronephrosis, and cleft lip and palate (Figures 1B–F).

**FIGURE 2:**
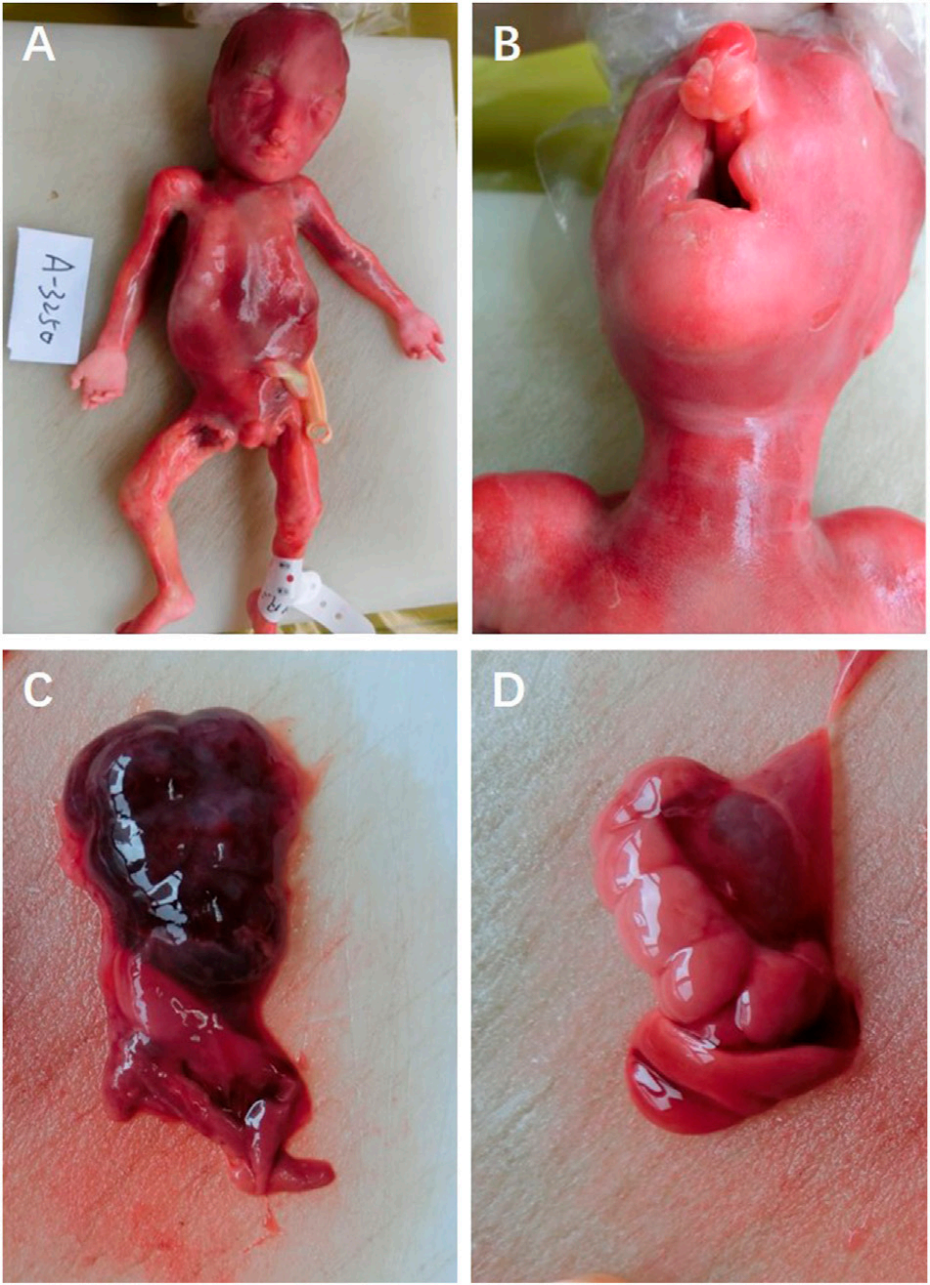
**(A)** Fetal appearance, **(B)** cleft lip and palate, **(C)** right renal dysplasia, and **(D)** hydronephrosis of the left kidney.

The parents opted to terminate the pregnancy *via* induction. Amniocentesis was performed to obtain the amniotic fluid prior to the intra-amniotic injection of rivanol solution after signing the informed consent, and they accepted the trio whole-exome sequencing (trio-WES) to identify the underlying etiology. No ectrodactyly or lobster-claw deformity was observed (Figure 2A). External examination showed no positive signs of ectodermal dysplasia (sparse hair, nail dysplasia, abnormal teeth, lacrimal duct obstruction, sweat gland dysplasia, etc.,) and was consistent with the gestational age of the fetus (Figure 2A). The autopsy confirmed bilateral cleft lip and palate (bilateral cleft lip, alveolar cleft, and complete cleft palate), right renal dysplasia, and hydronephrosis of the left kidney (Figures 2B–D).

**FIGURE 3:**
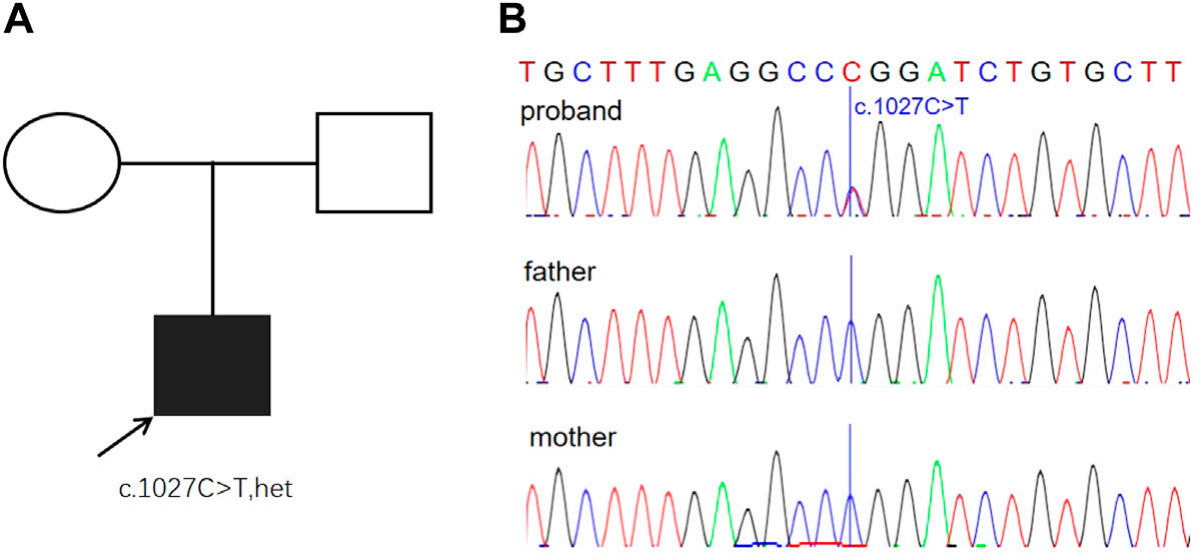
Genetic findings from the family. **(A)** Pedigree of the family with segregation of the identified TP63 mutation. The square represents male, and circles represent female. The filled symbol indicates the affected individual. **(B)** Variation of c.1027C > T is a *de novo* missense mutation (R304W) identified in the proband. The parents were tested and did not carry the mutation. Blue lines indicate the point mutation.

Different diseases were considered based on the aforementioned findings, including triploidy, microdeletion and microduplication syndromes, and single-gene disorders. To further search the underlying genetic causes, karyotyping and chromosomal microarray analysis (CMA) were performed, and both the results were normal (data not shown), suggesting no pathogenic copy number variations. The trio whole-exome sequencing (WES) was then performed, and a *de novo* missense mutation (c.1027C > T R304W) was detected in exon 8 of the *TP63* gene (Figure 3A). Nevertheless, none of the mutation at this site was found in the parents. Sanger sequencing confirmed this conclusion (Figure 3B). This mutation is pathogenic to EEC and had been demonstrated to disrupt the DNA-binding affinity of p63 and resulted in reduced transactivation activity (Browne et al., 2011).

## Discussion

EEC syndrome is a rare genetic disorder characterized by the following triad: 1) ectrodactyly, more precisely, limb malformations. The classic limb malformation is ectrodactyly, also called lobster claw, or split hands and/or feet, which is caused due to the lack of one or more central digits. Other limb deformities such as syndactyly, polydactyly, and abnormal size or shape of digital phalange of one or more fingers are also observed with an increasing number of literature studies being reported (Kumar et al., 2007; Koul et al., 2014). 2) Ectodermal dysplasia, which means the abnormal development of structures derived from the embryonic ectodermal layer. The common manifestations include sparse hair, nail dysplasia, abnormal teeth, lacrimal duct obstruction, and sweat gland dysplasia (Kumar et al., 2007; Goldsmith et al., 2014; Koul et al., 2014). 3) Cleft lip with or without palate (Iqbal Ali et al., 2013), which is often observed in patients with EEC syndrome.

Tumor protein p63 (TP63) encodes a high sequence homology of the p53 family of transcription factors, which is the master regulator during ectodermal and epidermal development. Disruption of TP63 in humans results in several overlapping disorders, such as ankyloblepharon-ectodermal defects cleft lip/palate (AEC) syndrome, acro–dermo–ungual–lacrimal–tooth (ADULT) syndrome, cleft lip/palate syndrome 3 (EEC3), limb-mammary syndrome, split-hand/foot malformation type 4 (SHFM4), isolated cleft lip/cleft palate (orofacial cleft 8), and Rapp–Hodgkin syndrome (RHS) (Rinne et al., 2007). The functional domain of TP63 contains an N-terminal transactivation domain, a central DNA-binding domain (DBD), an oligomerization domain, a sterile-alpha motif domain, and a C-terminal transactivation inhibitory domain. Several isoforms with different N-terminal ends are found with distinct biological properties (Bourdon, 2007). TAp63α, which represents the full gene product, is expressed in oocytes (Suh et al., 2006), while ΔNp63α, which lacks the N-terminal transactivation domain, is the major isoform expressed in the epidermis (Yang et al., 1999). The ratio of ΔNp63 and TAp63 isoforms may govern the maintenance of epithelial stem cell compartments and regulate the initiation of epithelial stratification from the undifferentiated embryonal ectoderm (Straub et al., 2010). EEC is mainly caused by point mutations in the DBD, which may impair the p63 protein binding to DNA. Five frequently mutated amino acids (R204, R227, R279, R280, and R304), which are all located in the CpG islands, explain almost 90% of the EEC patients (Brunner et al., 2002).

Remarkably, p63 mutations in a mouse model result in an EEC syndrome-like phenotype [20.22]. P63-deficient mice lack all squamous epithelia and their derivatives, including hair, whiskers, teeth, and the mammary, lacrimal, and salivary glands (Mills et al., 1999; Yang et al., 1999). Particularly striking are severe limb truncations with forelimbs showing a complete absence of the phalanges and carpals and variable defects of ulnae and radiae and hindlimbs that are lacking altogether. Similarly, ECC syndrome patients manifest as a generalized ectodermal dysplasia (which presents as sparse hair, dry skin, pilosebaceous gland dysplasia, lacrimal duct obstruction, and oligodontia) and classic limb malformation (which presents as the lack of one or more central digits). Additionally, the truncated secondary palate and hypoplastic maxilla and mandibula in p63-deficient mice correspond to cleft lip with or without the cleft palate in EEC syndrome patients. Thus, the heterozygous mutation of TP63 plays an important role in EEC syndrome, whereas the genetic basis underlying the variable expressivity and incomplete penetrance of EEC remains unclear. VernerssonLindahl et al. (2013) discovered that clefting and skin defects are caused by loss of Trp63 function, while limb anomalies are due to gain- and/or dominant-negative effects of Trp63 by utilizing two mouse models with alleles encoding Trp63R279H (Trp63Aam1-R279HN and Trp63Aam2-R279H). Furthermore, their studies identified TAp63 as a strong modifier of EEC-associated phenotypes, with regard to both penetrance and expressivity. At present, no specific molecular pathway has been reported to be associated with genitourinary anomalies caused by the p63 mutation. Nardi et al. (1992) speculated that the altered gene in EEC syndrome affected the ectoderm and its derivatives, leading to the two fundamental types of GU defects: abnormal glandular urethral development causing hypospadias and anomalous genesis of the ureteric bud.

Remarkable clinical variability has been observed for different TP63 mutations. Regarding the special amino acid mutation of R304 observed in the present case, we summarized the clinical presentations of the reported 33 cases with EEC syndrome and the R304 mutation (Table 1) (Wessagowit et al., 2000; van Bokhoven et al., 2001; Hamada et al., 2002; de Mollerat et al., 2003; Dianzani et al., 2003; Paranaíba et al., 2010; Gawrych et al., 2013; Okamura et al., 2013; Brueggemann and Bartsch, 2016; Wenger et al., 2018). Orofacial cleft, the most significant feature of EEC syndrome, is presented in approximately 80% of patients. Most of the patients presented with cleft lip and palate (26/33), while only three cases are observed with isolated cleft lip or isolated cleft palate (Hamada et al., 2002; de Mollerat et al., 2003; Okamura et al., 2013). Among limb malformations, the penetrance of ectrodactyly, syndactyly, oligodactyly, and polydactyly are 63%, 57%, 14%, and 11% respectively. The ectodermal dysplasia of nail, lacrimal, hair, and teeth quite commonly occurs in over 40% of EEC cases. Genitourinary anomalies, including the kidney and urinary problems, were reported in 28% (10/33). Conductive hearing loss is not scarce and was detected in about 20% (7/33). Strikingly, neurodevelopment disorders, such as intellectual disability and speech delay, were reported in a relatively high percentage of 20% (7/33), which is higher than other under-recognized features.

**TABLE 1:** Phenotypic characteristics of EEC syndrome associated with R304 mutations.

	Family	Patient	Confirmed *de novo*	Mutation		Limb deformity^a^	Facial clefting^b^	Ectodermal dysplasia^c^	Others^d^
				Nucleotide	Amino acid				
Wessagowit et al. (2000)	1	1	1	C1027T	R304W	E (1) and S (1)	L (1) and P (1)	H (1), N (1), T (1), and ND (1)	G (1)
van Bokhoven et al. (2001)	2	3	1	C1027T	R304W	E (1) and S (2)	L (3) and P (3)	H (3), S (2), N (3),T (2), and L (3)	K (2) and HL (2)
	5	5	4	G1028A	R304Q	E (5) and S (1)	L (4) and P (4)	H (5), S (2), N (3), T (4), and L (5)	K (2) and G (1)
Hamada et al. (2002)	1	1	1	C1027T	R304W	E (1) and S (1)	—	N (1) and ND (1)	—
	5	6	5	G1028A	R304Q	E (5), S (3), O (2), and P (1)	L (3) and P (4)	H (2), S (2), N (2), T (1), ND (3), and L (2)	HL (4), U (3), ID (2), AF (1), and K (1)
de Mollerat et al. (2003)	1	1	0	C1027T	R304W	E (1) and S (1)	L (1) and P (1)	H (1) and N (1)	—
	2	2	0	G1028A	R304Q	E (2) and S (1)	L (2) and P (2)	H (2), N (2), T (2), and L (2)	—
	1	1	0	G1028C	R304P	E (1), S (1), and O (1)	L (1)	H (1) and T (1)	ID (1) and BL (1)
Dianzani et al. (2003)	1	5	0	G1028A	R304Q	E (1), S (4), O (1), and P (3)	L (5) and P (5)	H (1), N (5), and L (5)	—
Paranaíba et al. (2010)	1	1	1	C1027T	R304W	E (1)	L (1) and P (1)	H (1), S (1), N (1), T (1), and L (1)	ID (1) and HL (1)
Okamura et al. (2013)	1	1	0	G1028A	R304Q	E (1) and S (1)	P (1)	H (1), N (1), T (1), and L (1)	—
Gawrych et al. (2013)	1	1	0	C1027T	R304W	S (1) and O (1)	L (1) and P (1)	H (1), S (1), and ND (1)	SD (1)
Brueggemann and Bartsch (2016)	1	2	0	G1028A	R304Q	E (1) and S (1)	L (2) and P (2)	H (1), S (1), and N (1)	—
Wenger et al. (2018)	1	1	1	C1027T	R304W	—	L (1) and P (1)	N (1) and ND (1)	K (1) and U (1)
	1	2	0	G1028A	R304Q	E (1) and S (2)	L (2) and P (2)	N (2), T (2), ND (2), and L (2)	K (2), U (2), and SD (1)
Total	25	33	14	—	—	E (22), S (20), O (5), and P (4)	P (28) and L (27)	N (25), L (21), H (20), T (15), ND (9), and S (9)	K (8), HL (7), U (6), ID (4), SD (2), and G (2)

^a^ E, ectrodactyly; O, oligodactyly; P, polydactyly; S, syndactyly.

^b^ L, cleft lip; P, cleft palate.

^c^ H, hair; L, lacrimal ducts; N, nail; ND, nasolacrimal duct; S, skin; T, teeth.

^d^ BL, blindness; G, genital deformities; HL, hearing loss; K, kidney malformations; ID, intellectual disability; SD, speech delay; U, urinary tract anomalies.

The prenatal spectrum of EEC fetuses is quite different from that of postnatal cases. This is mainly because ectodermal dysplasia, the notable characteristic in most postnatal EEC cases, could not be detected by antenatal ultrasound. In addition, the incomplete penetrance makes it even harder for prenatal diagnosis. To further delineate the prenatal spectrum of EEC syndrome, we summarized the prenatal EEC cases from the literature. Up to now, only 14 fetal cases have been reported, including our fetus (not limited to the R304W mutation) (Supplementary Table S1) (Witters et al., 2001; Hamada et al., 2002; Janssens et al., 2008; Simonazzi et al., 2012; Gawrych et al., 2013; Enriquez et al., 2016; Hyder et al., 2017; Yang et al., 2017; Wenger et al., 2018; Liu et al., 2019; Friedmann et al., 2020). Cleft lip and palate is the most common prenatal ultrasound finding with the highest incidence of 64.3% (9/14). It would be higher if the autopsy results are accounted. To our surprise, the kidney malformations, including hydronephrosis and cystic dysplasia, rank the second with a percentage of 57.1% (8/14). The prevalence of genitourinary abnormalities in postnatal cases is much lower than that in prenatal cases, which might be due to the lack of renal ultrasound scans in postnatal patients.

Genitourinary abnormalities are an under-recognized feature of EEC in prenatal cases. There are very few recorded cases available on the prenatal ultrasound outcomes in patients with EEC, while most studies report fetuses with classic findings of cleft lip and palate or clefting of the hands and feet. Several studies have focused on the prenatal outcomes in patients with genitourinary abnormalities. Here, we report a fetus with cleft lip and palate, right polycystic kidney, and fetal megacystis (first trimester) and identify a pathogenic variation of the *TP63* gene by WES associated with EEC syndrome. To the best of our knowledge, this is the first report in China that described a fetus who went on to receive a diagnosis of prenatal EEC with genitourinary anomalies (right polycystic kidney and fetal megacystis). In our case, the earliest finding was an enlarged bladder with the diameter of 12 mm in the first trimester. Although the diameter of the bladder decreased gradually into a normal size, the fetus was finally diagnosed as the right multicystic kidney and left hydronephrosis. In other world countries, similarly, there are a less number of reports. Enriquez et al. (2016) reported a similar EEC fetus with unusual bladder distension at 14 weeks of gestation, who was finally confirmed to have multiple abnormalities of the lower genitourinary tract after autopsy. Janssens et al. (2008) also reported a fetus of EEC syndrome with bladder distension, mild bilateral hydronephrosis, and a prune belly. Our case presented with cleft lip and palate, right polycystic kidney, and fetal megacystis. Considering the solitary cleft lip and palate, negative family history, and lack of clefting of the hands and feet, the diagnosis of EEC would have been quite difficult without WES. In our case, an abnormal enlarged bladder in the first trimester would imply the possibility of genitourinary abnormalities. We suggest that physicians should alert the occurrence of EEC syndrome when genitourinary anomalies are observed with orofacial cleft or other findings. Prenatal WES is very useful to find the true etiology when negative results are presented in karyotyping and CMA.

## Conclusion

Prenatal diagnosis of some certain monogenetic disorders has long been proved difficult due to very few cases and limited ultrasound phenotypes. We reported a fetus of EEC syndrome with genitourinary anomalies, cleft lip and palate, and no symptoms of ectrodactyly or syndactyly. To the best of our knowledge, this is the first report in China that described a fetus who went on to receive a diagnosis of prenatal EEC with genitourinary anomalies (right polycystic kidney and fetal megacystis). Furthermore, a compilation of the prenatal manifestations of EEC is provided through a literature review, which can provide convenience for clinical applications. High penetrance is observed both in orofacial cleft and genitourinary malformations, while genitourinary anomalies are usually an under-recognized feature of EEC. Our case emphasizes the phenotypic spectrum of *TP63*-related disorders to include polycystic kidneys and fetal megacystis in the first trimester as a prenatal feature of EEC. Therefore, EEC syndrome should be suspected when patients have genitourinary anomalies with orofacial cleft or limb deformities. Finally, our report also reinforces the recommendation that the application of WES could help clarify unexpected prenatal findings, significantly improve the accuracy of prenatal diagnosis of genitourinary anomalies, and add prenatal phenotypic characteristics to known single-gene disorders.

## Data Availability

The datasets for this article are not publicly available due to concerns regarding participant/patient anonymity. Requests to access the datasets should be directed to the corresponding authors.
