# Supplementary material for: Case report: Prenatal diagnosis of Ectrodactyly–Ectodermal dysplasia–Cleft syndrome (EEC) in a fetus with cleft lip and polycystic kidney
Source: Front Genet. 2022 Oct 31;13:1002089. doi: 10.3389/fgene.2022.1002089 (PMC9662688; doi:10.3389/fgene.2022.1002089)
Supplement: Supplementary file 1 [file Table1.docx]

Supplementary Table S1 Clinical presentations of prenatal findings of TP63-related disorders

|  | Gestation | Inheritance | Mutation | | Limb deformity | Facial clefting | Others | Postnatal or autopsy findings |
| --- | --- | --- | --- | --- | --- | --- | --- | --- |
|  |  |  | Nucleotide | Amino acid |  |  |  |  |
| Witters et al. 2001[34] | 16 | paternal | A577G | K193E | ectrodactyly | - | - | - |
| Hamada et al. 2002[26] | 22 | - | G1028A | R304Q | - | - | oligohydramnios,  severe urogenital abnormalities | Multiple renal dysplasia,  ectrodactyly |
| Janssens et al. 2008[35] | 15 | maternal | C610T | Q204W | - | CP | bladder distension, bilateral hydronephrosis | CLP, ectrodactyly, bilateral hydronephrosis |
| Simonazzi et al. 2012[36] | 19 | de novo | A598G | K161E | ectrodactyly | CLP | oligodactyly | syndactyly |
| Gawrych et al. 2013[31] | 20 | - | C1027T | R304W | - | CLP | oligodactyly | syndactyly, sparse hair and speech delay |
| Enriquez et al. 2016[37] | 18 | - | G1051A | D351N | - | - | megaureters with marked bilateral hydronephrosis | CLP, ectrodactyly, kidney and urinary tract anomalies |
|  | 14 | - | G1051A | D351N | - | - | unusual bladder distension | CLP, retrognathia, low-set ears, and hypotelorism. Multiple abnormalities of the lower genitourinary tract |
| Hyder et al. 2017[9] | 20-30 | - | A740T | H247L | - | CLP | bilateral hydronephrosis and megaureter | - |
| Yang et al. 2017[38] | 19 | maternal | C955T | R280C | ectrodactyly | CLP | oligodactyly | - |
| Wenger et al. 2018-twin1[33] | - | de novo | C1027T | R304W | - | CLP | - | CLP, absence lacrimal ducts, limb deformities |
| Wenger et al. 2018-twin2 | - | de novo | C1027T | R304W | - | CLP | - | CLP, absence lacrimal ducts, limb deformities |
| Liu et al. 2019[39] | 25 | de novo | C952T | R318T | - | CLP | right kidney cystic dysplasia | - |
| Friedmann et al. 2020[40] | 22 | de novo | A740G | H247R | - | - | multicystic dysplastic kidneys | Kidney abnormalities and limb deformities |
| Our case | 22 | de novo |  | R304W | - | CLP | right multicystic kidney and left hydronephrosis | CLP, right multicystic kidney and left hydronephrosis |

CP: cleft palate; CLP: cleft lip and palate
